# Benefits of Anthocyanin-Rich Black Rice Fraction and Wood Sterols to Control Plasma and Tissue Lipid Concentrations in Wistar Kyoto Rats Fed an Atherogenic Diet

**DOI:** 10.3390/molecules25225363

**Published:** 2020-11-17

**Authors:** Aneta Kopeć, Jerzy Zawistowski, David D. Kitts

**Affiliations:** 1 Department of Human Nutrition and Dietetics, Faculty of Food Technology, University of Agriculture in Krakow, Balicka 122, 31-149 Kraków, Poland; aneta.kopec@urk.edu.pl; 2 Faculty of Land and Food Systems, University of British Columbia 209-2205 East Mall, Vancouver, BC V6T 1Z4, Canada; jerzy.zawistowski@ubc.ca

**Keywords:** black rice cyanidin-3-*O*-glucoside, wood sterols, dyslipidemia, CVD

## Abstract

Background**:** This study reports on the relative effects of administrating a cyanidin-3-*O*-glucoside-rich black rice fraction (BRF), a standardized wood sterol mixture (WS), and a combination of both to lower plasma and target tissue lipid concentrations in Wistar Kyoto (WKY) rats fed atherogenic diets. Methods**:** Male WKY (*n* = 40) rats were randomly divided into five groups, which included a nonatherogenic control diet and atherogenic diets that included a positive control and atherogenic diets supplemented with BRF or WS, respectively, and a combination of both BRF + WS. Plasma and target tissue liver, heart and aorta cholesterol, and triacylglycerides (TAG) content were also measured. Results**:** Rats fed atherogenic diets exhibited elevated hyperlipidemia compared to counterparts fed nonatherogenic diets (*p* < 0.001); this effect was mitigated by supplementing the atherogenic diets with BRF and WS, respectively (*p* < 0.05). Combining BRF with WS to enrich the supplement lowered cholesterol similar to the WS effect (*p* < 0.05) and lowered TAG characteristic to the BRF effect (*p* < 0.05). Conclusions**:** Rats fed diets containing BRF or WS effectively mitigate the hypercholesterolemia and elevated TAG induced by feeding an atherogenic diet. The benefit of adding BRF + WS together is relevant to the lipid parameter measured and is target tissue-specific.

## 1. Introduction

Cardiovascular disease (CVD) represents a major cause of death in Europe, North America, and in some parts of Asia [1,2,3,4]. According to the World Health Organization 2016 records, CVD accounted for 31% of the total global mortality [5]. This knowledge has empowered today’s consumers to be selective at identifying foods that have both adequate essential nutrient contents to meet daily requirements while also providing distinct health benefits attributed to plant-based bioactives that can protect against CVD. Examples of bioactive food constituents that lower serum cholesterol are plant sterols [6,7,8] and, also, pigmented anthocyanins that display antioxidant and anti-inflammatory properties [9,10,11,12], the combination of which could have a greater role in protecting against CVD. Hypercholesterolemia, along with a disturbed antioxidant status and onset of inflammation, are major underlying causes of CVD.

**Figure 1:**
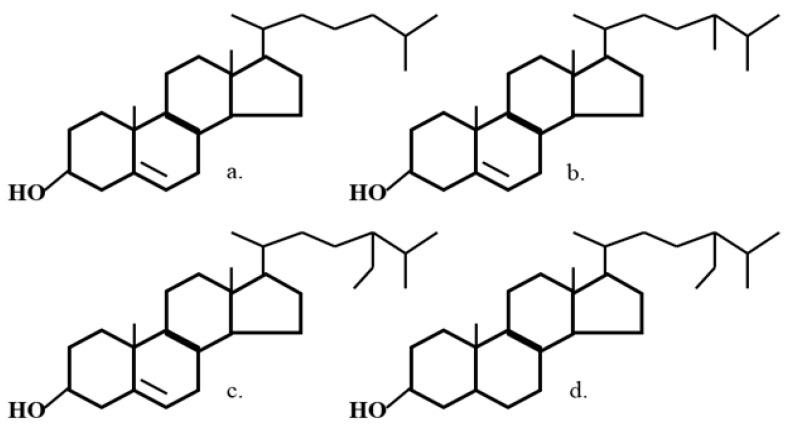
Chemical structures of (**a**) cholesterol, (**b**) campesterol, (**c**) β-sitosterol, and (**d**) β-sitostanol.

Phytosterols are natural ingredients present in vegetable oils and, also, can be recovered from wood sources. Phytosterol intake from a traditional Western diet depends on personal habits and geographical location [13]. A typical Western diet contains about 300 mg/day of phytosterols [13,14,15]. Plant sterols and stanols are structurally similar to cholesterol (Figure 1) [16] and are effective at lowering the plasma total and low-density lipoprotein cholesterol [7,17,18].

**Figure 2:**
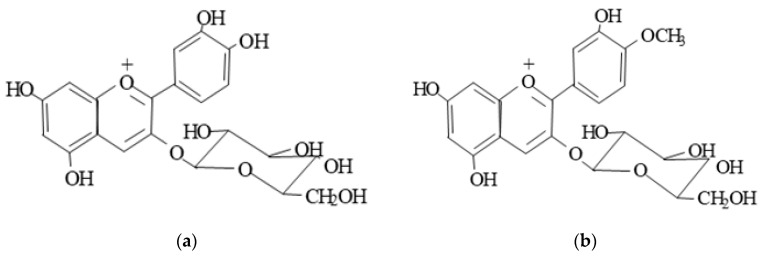
Structures of cyanidin-3-*O*-glucoside (**a**) and peonidin-3-*O*-glucoside (**b**).

Black rice, on the other hand, is an excellent source of anthocyanidins, in addition to dietary fiber, flavonoids, and other polyphenols. Rice oil contains nonatherogenic fatty acids [19,20] and is also a good source of plant sterols, such as oryzanol. These bioactive components are unsaponifiable, nonglyceride components [20], which contribute to cholesterol-lowering effects reported in many animal and human studies [19,20,21,22,23,24]. The pigment from black rice contains two major anthocyanins—namely, cyanidin-3-*O*-glucoside and peonidin-3-*O*-glucoside—the former being predominant [10,23,25] (Figure 2).

Anthocyanins are naturally occurring phenolic compounds that provide color and bioactive properties, such as antioxidants [10] and lowering of serum cholesterol and triacyglycerides in rats, when fed to rats on a daily basis [26].

There are no studies that have examined the effects of consuming a combination of these bioactive agents to mitigate the changes of known CVD risk factors induced by feeding on an atherogenic diet. The objective of the present study was to demonstrate a potential interaction or added effect of combining a standardized cyanidin-3-*O*-glucoside black rice fraction (BRF) and a known wood sterol (WS) mixture to protect against elevated serum lipids and, moreover, target tissue cholesterol deposition in rats fed an atherogenic diet.

## 2. Results

### 2.1. Body Weight Gain and Heart and Liver Weights

**Figure 3:**
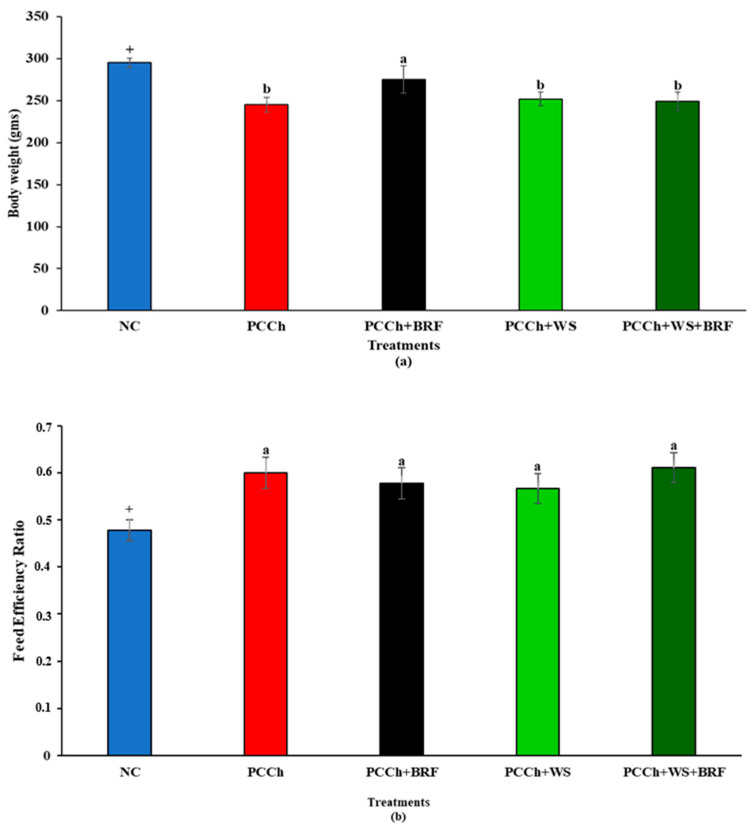
(**a**) Body weight gain of experimental rats. NC- AIN-76A diet. PCCh positive control (0.5% cholesterol, 0.05 cholic acid, 3% butter). BRF-black rice fraction. PCCh +BRF positive control with black rice fraction. PCCh +WS positive control with wood sterols. PCCh +WS+BRF positive control with wood sterols and black rice fraction. Bars represent means SEM/ (n = 8). The + symbol signifies a significant difference (*p* < 0.05) between the negative cholesterol (NC) and positive control group (PCCh). Bars with different superscript letter (a,b) are significantly different (*p* ≤ 0.05).Bars with different superscript letter (a,b) are significantly different (*p* ≤ 0.05). (**b**). Fed efficiency ratio of rats. NC- AIN-76A diet. PCCh positive control (0.5% cholesterol, 0.05 cholic acid, 3% butter). BRF-black rice fraction. PCCh + BRF positive control with black rice fraction. PCCh + WS positive control with wood sterols. PCCh + WS + BRF positive control with wood sterols and black rice fraction^1.^ Bars represent means SEM/ (n = 8). The + symbol signifies a significant difference (*P* < 0.05) between the negative cholesterol (NC) and positive control group (PCCh).

The body weight gain (Figure 3a) of rats fed experimental atherogenic diets with BRF was significantly higher (*p* < 0.05) compared to other experimental groups, but no differences were observed for the feed efficiency ratio (FER, Figure 3b) between groups. Supplementary Table S1 presents the food intake and tissue organ weights, respectively, of animals fed on atherogenic experimental diets for 11 weeks. We also include data from rats fed a negative cholesterol (NC) diet for comparison of the plasma and tissue lipid concentrations typical of an AIN-76 A control diet. The atherogenic diets supplemented with WS and a combination of WS + BRF lowered the liver weight significantly (*p* < 0.05). The heart weight did not vary significantly between treatments. Feeding atherogenic diets with and without WS and BRF supplements did not affect the hematocrit or hemoglobin concentrations.

### 2.2. Blood Constituent Analysis 

There was no dietary effect observed that indicated differences in the plasma glucose levels of animals fed the control or experimental diets, respectively. Similarly, the plasma Oxygen Radical Absorbance Capacity (ORAC) and C-Reactive Protein (CRP) were not different between animals fed different diets (data not shown). This result may be explained by the fact that only one-time blood sampling of the blood was made at the time of sacrifice, which was likely insufficient to show meaningful trends in these biochemical parameters. 

**Figure 4:**
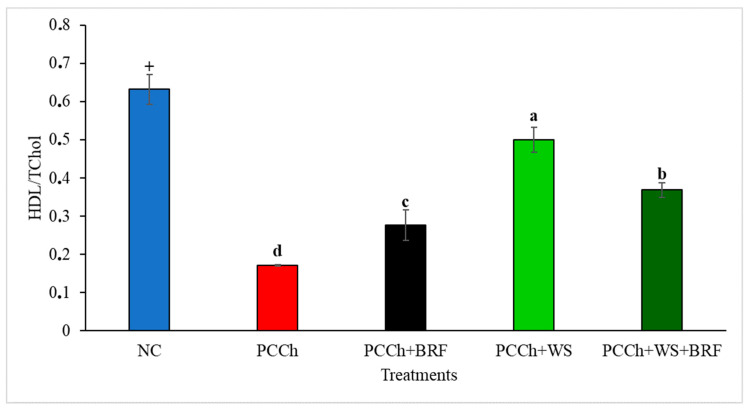
Plasma high-density lipoprotein (HDL) cholesterol/total cholesterol ratio in experimental groups. NC: AIN-76A diet. PCCh: positive control (0.5% cholesterol, 0.05 cholic acid, 3% butter). BRF: black rice fraction. PCCh + BRF: positive control with black rice fraction. PCCh + WS: positive control with wood sterols. PCCh + WS + BRF: positive control with wood sterols and black rice fraction. Bars represent means ± SEM (*n* = 8). The + symbol signifies a significant difference (*p* < 0.05) between the negative cholesterol (NC) and positive control groups (PCCh). Bars with different superscript letters (a, b, c, and d) are significantly different (*p* ≤ 0.05).

Animals fed on the experimental atherogenic diets (PCCh) exhibited hyperlipidemia, compared to counterparts fed a NC diet. The total plasma cholesterol concentration (TC) was significantly (*p* < 0.05) lowered in animals fed on experimental PCCh diets that also contained BRF and WS, respectively. The relative degree of lowering was greatest in animals fed on the WS atherogenic diet. Combining BRF + WS in atherogenic diets resulted in changes in the plasma cholesterol that resembled animals fed the WS diet (Table 1). The high-density lipoprotein (HDL)/TC ratio was significantly higher (*p* < 0.05) for all animals fed experimental PCCh diets that included BRF, WS, or the combination of BFR + WS, in comparison to the control PCCh diet (Figure 4). Rats fed the nonatherogenic control diet also exhibited a distinct high-plasma HDL/TC ratio. This observation can be attributed to the fact that the HDL cholesterol concentrations were significantly (*p* < 0.05) higher in WS fed rats on the atherogenic diet (Table 1). Plasma non-HDL cholesterol concentrations were significantly (*p* < 0.001) lower in rats fed atherogenic diets containing BRF, but the greatest reductions were obtained in counterpart animals fed on WS (Table 1). This observation led to the conclusion that the principle component for the cholesterol-lowering effect observed in rats fed on the BRF + WS diet (*p* < 0.05) was indeed attributed to the presence of WS more so than BRF. This trend was reversed for the plasma triacylglyceride (TAG) responses in rats fed the atherogenic diets supplemented with BRF. For example, plasma TAG levels were significantly (*p* < 0.01) lower in rats fed diets with added BRF and lower than those fed WS (*p* < 0.05). Of interest was the observation that the combination of adding BRF + WS produced a significantly lower (*p* < 0.05) plasma TAG level compared to rats fed only WS (Table 1). Hence, rats fed on atherogenic diets that contained both BRF + WS exhibited plasma TAG concentrations that resembled animals fed the BRF supplement. These trends were not observed for plasma phospholipids responses in rats fed the combination of BRF + WS but, rather, resembled moreso the WS group (Table 1).

### 2.3. Tissue Lipid Concentrations

The crude lipid concentration in liver tissue was significantly (*p* < 0.05) higher in rats fed the atherogenic diet that did not contain added BRF and WS bioactives, respectively (Table 2). The greatest relative reduction in liver cholesterol of rats fed atherogenic diets was observed in rats fed on the WS and BRF + WS diets, respectively (*p* < 0.05) (Table 2). The presence of both BRF + WS to significantly reduce liver (*p* < 0.05) cholesterol in these animals was therefore attributed mostly to the presence of WS in the phytochemical supplement mixture. In addition, the lowest liver triacylglycerol concentrations were observed in rats fed on diets with BRF (*p* < 0.05), which resembled counterparts fed the atherogenic BRF + WS diet. 

In heart tissue, cholesterol accumulation in animals fed atherogenic diets was higher (*p* < 0.05) compared to NC diet-fed control animals. The only exception to this was with the atherogenic rats supplemented with BRF, which exhibited characteristically low heart cholesterol accumulation compared to the WS counterparts (*p* < 0.05) and more closely resembled the NC controls. The presence of both BRF and WS in the atherogenic diet was not sufficient to reduce heart cholesterol to the level observed for rats fed the BRF-only diet. Of interest was the fact that measuring the cholesterol content in extracted aortic tissue produced dramatically different results. Rats fed the atherogenic diet produced a five-fold increase in aortic cholesterol content compared to the NC diet-fed animals. The low aortic cholesterol concentration found in NC controls was also observed in animals fed on the BRF atherogenic diet, but this was dramatically lower (*p* < 0.05) compared to counterparts fed on the WS atherogenic diet. Combining both BRF and WS supplements in the atherogenic diet indeed produced a lower cholesterol accumulation (*p* < 0.05) compared to the atherogenic control, but the extent of the cholesterol reduction did not match what was obtained when WS and BRF were fed individually (Table 2).

## 3. Discussion

The present study shows the relative efficacy of supplementing experimental diets that are formulated to hyperlipidemic, with a standardized anthocyanin-rich BRF and sterol-rich WS, to characteristically mitigate both plasma and tissue lipid increases that are attributable risk factors for CVD. Moreover, in some specific instances, the combination of having both BRF + WS included in the supplemented diet produced an effect that was more or less typical of BRF or WS, added individually, but this was dependent on the plasma lipid measurement and the specific tissue sampled.

In this study, adding 1% BRF to the atherogenic diet produced a greater weight gain in rats, indicating a total acceptance of the diet that included BRF. Other workers reported weight loss in mice and rabbits when fed on BRF-containing diets [9,27]. BRF has been shown to have nutritional qualities outside their antioxidant capacity that include high calcium and protein bioavailabilities [21,27]. Animals fed atherogenic diets that were formulated to contain added cholesterol and lard as the fat source, along with the defined anthocyanin mixture that composed BRF supplementation, showed a lower body weight gain reported by others [28]. In contrast, other studies reported no change in body weight gain in a variety of different experimental animals fed atherogenic diets that included BRF [29,30] or plant sterols [31].

The atherogenic diets fed to rats in this study were composed of butter and canola oil, along with cholesterol and cholic acid. The observed greater liver cholesterol content of control rats corresponded to the noted hypercholesterolemia that occurred in these animals. Supplementing the atherogenic diets with BRF and WS produced lower liver weights, respectively. We attribute the effect associated with BRF to the fact that it is a good source of soluble dietary fiber and, also, contains trace amounts of oryzanol. These bioactive constituents are well-known to form complexes with cholesterol directly in the intestine and, also, bind to bile acids that are required for cholesterol absorption, the net effect being reduced cholesterol absorption that promotes excretion. Oryzanol, a phytosterol also recovered from the black rice outer layer [32], is also known to be effective at reducing cholesterol absorption and increasing the excretion of bile acids [33], thus potentially contributing to lower liver weights of BRF-fed rats.

Similarly, the higher total plasma cholesterol concentrations observed in rats fed on the atherogenic control diet, compared to the NC diet-fed rodents, were reduced when the atherogenic diets were supplemented with BRF and WS, respectively. Similar results have been reported with black rice in C57BL/6J mice fed a high-fat diet containing an anthocyanin-rich extract derived from a similar rice source [9]. Frequent exposure to anthocyanins has been associated with the stimulation of transporters that involve anthocyanin absorption [34]. Thus, daily consumption could potentially decrease CVD risk, associated with conditions of inflammation and atherosclerosis [9,10,24,35,36]. Black rice and outer layer fractions also significantly reduce serum lipids [35] and atherosclerotic plaque formation in hypercholesterolemic rabbits and Apo-E-deficient mice [9,22,27]. These authors reported that the supplementation of experimental diets with the black rice outer layer fraction decreased the level of LDL cholesterol in Apo-E-deficient mice. This observation was not confirmed in other studies with rabbits fed black rice [27]. On the other hand, rats fed diets containing WS alone showed a greater reduction in plasma cholesterol compared to those fed diets containing only BRF. The primary mechanism for the hypocholesterolemic effect attributed to phytosterol intake is related to the inhibition of the micellar cholesterol solubilization of cholesterol, which results in reduced cholesterol absorption from the intestinal lumen and promotes excretion [18,37,38]. The consumption of about 2 g of sterols per day reduces LDL cholesterol by 8–15% and, consequently, reduces the risk of cardiovascular disease in humans [39]. Currently, also, the anti-inflammatory effect of plant sterols is possible; albeit, these data are inconsistent [38,40,41]. The mechanism for WS lowering plasma cholesterol is well-defined to include a competition to form mixed micelles that are required for cholesterol absorption in the small intestine, due to the similarities in structure. The limited solubility of WS in the intestinal chyme also interferes with the solubility of cholesterol, which, in turn, contributes to coprecipitation in the intestinal lumen and low cholesterol bioavailability [18]. WS also competes with cholesterol for various transporters at the apical surface of enterocytes—namely, Niemann-Pick C1-like 1 protein (NPC1L1) and adenosine triphosphate (ATP) cassette protein-binding transporters (ABCG5/G8), which regulate the influx and efflux of cholesterol and sterols between the intestinal lumen and the intestinal mucosa [41]. The greater decrease in plasma cholesterol in animals fed diets supplemented with a combination of WS and BRF paralleled a similar reduction in the total cholesterol contents of the liver, heart, and aorta tissues. For the most part, the effect on cholesterol lowering was most dramatic when WS were present in the atherogenic diet. This was also true when both BRF + WS were combined, with the expected added effect to reduce cholesterol even further not realized but, rather, attributed mainly to the presence of WS in the mixture. However, having BRF present in the mixture may be contributed, to some extent, by other mechanisms due to the presence of both anthocyanins and dietary fiber. In addition to the reported benefits of having the antioxidant activity of anthocyanins to protect against cardiovascular diseases [42,43], these compounds also inhibit rate-limiting enzyme activities for cholesterol synthesis—namely, 3-hydroxy-3-methyl-glutaryl-coenzyme A (HMG-CoA) and cholesteryl ester transfer protein (CEPT) enzymes, which are linked to changes in endogenous cholesterol synthesis [43,44,45]. Pong et al. [46] showed that mulberry extracts contain anthocyanins, decreased hepatic lipids, and reduce the level of HMG-CoA reductase in hamsters. A similar cascade of lipid specific metabolic reactions were suppressed in rats fed anthocyanin-rich juice [26]. The soluble fiber present in back rice also has cholesterol-lowering properties [47,48], attributed to a reduced dietary cholesterol bioaccessibility for absorption. Moreover, soluble dietary fiber derived from BRF could also be a substrate for bifidobacteria fermentation in the colon, with a subsequent production of short-chain fatty acids (SFCA) that may suppress cholesterol biosynthesis, thus further contributing to low cholesterol levels in plasma and tissue [49,50].

The presence of WS, BRF, or the combination of both in experimental atherogenic diets fed to rats increased the plasma HDL cholesterol. Great interest exists in the strong inverse and independent relationship beween HDL and clinical atherosclerosis [49]. HDL transports cholesterol or cholesterol esters from peripheral tissues to the liver, where cholesterol is metabolized and transformed into bile acids; thus, this route of cholesterol disposal has an important role in protecting against atherosclerosis [27,49]. Researchers have shown that plant sterols slightly increase plasma HDL by elevating a specific subfraction of HDL, HDL3. The mechanism is not completely elucidated and may depend on many related factors, such as the composition of the food matrix and baseline blood lipid profile of the clinical subjects [18]. Anthocyanins may also contribute to increasing the level of HDL cholesterol by the inhibition of cholesteryl ester transfer proteins [51]. On the other hand, plasma non-HDL cholesterol levels were reduced in rats fed on atherogenic diets that contained both BRF and WS. LDL, a well-documented risk factor for cardiovascular disease, is involved in the transportation of cholesterol or cholesterol esters from the liver to peripheral tissues. Increasing the hepatocyte LDL receptor activity due to the presence of dietary WS and BRF would result in an increased influx of cholesterol from the plasma to the hepatocyte, thereby resulting in an increased compartmentalization of cholesterol that would lower both the plasma non-HDL cholesterol and total cholesterol concentrations.

Our results showing BRF to reduce serum TAG concentrations are supported by other studies that demonstrated bioactive constituents present in rice were involved in TAG digestion and metabolism [23]. Our finding that the presence of BRF to lower plasma triacylglyceride levels in rats was more effective than adding WS could be related to the dietary fiber content, which effectively increases the gut transit time and, as consequence, the degree of contact with digestive enzymes such as pancreatic lipase. Studies conducted in rabbits fed a black rice outer layer reported no changes in the plasma triacylglycerols concentrations [27]. Nevertheless, apo E-deficient mice fed on an anthocyanin-rich BRF for 20 weeks exhibited reductions in serum TAG content [23]. Roohinejad et al. [52] also reported a similar finding in rats fed on pregerminated brown rice, and Ausman et al. [20] reported that rice bran oil was effective at decreasing plasma triacylglyceride (TAG) in hypercholesterolemic hamsters. Our study also showed that feeding WS effectively increased plasma TAG. This result agrees with Pritchard et al. [31] in studies conducted with apo E-deficient mice fed on an atherogenic diet containing WS. Vaskonen et al. [37] reported no significant changes in the TAG level in plasma-obese Zucker rats fed WS. Human clinical studies that administrated sterols in an encapsulated form at intakes of 1.8 g/day for four weeks reported a 7.4% decrease in plasma TAG in hypercholesterolemic subjects [47].

Although anthocyanins present in BRF possess antioxidant and anti-inflammatory activities [10,24,43,45], this was not an apparent factor in our experimental results. CRP is an early biomarker for inflammation and a good indicator of atherosclerosis risk [53]. Ma et al. and [54] and Oi et al. [55] showed that dietary fiber decreases the concentration of CRP in humans. Moreover, Devaraj et al. [38] reported that the consumption of orange juice with added WS for eight weeks resulted in lower serum CRP levels in human subjects. A meta-analysis by Rocha and coworkers [8] showed that the CRP level was not affected by WS in diets in various human studies. Unfortunately, our sampling protocal may not have enabled us to detect possible differences in CRP between rats fed different experimental diets. Future studies are required to include more frequent blood sampling to determine if, indeed, the inclusion of BRF and WS in atherogenic diets can promote a positive effect on CRP.

## 4. Materials and Methods

### 4.1. Animals and Diets

Wistar Kyoto (WKY) male rats, all 5 weeks of age, were purchased from Charles River, Montreal, PQ, Canada. Animals were acclimatized for 1 week on standard lab chow (Ralston Purina, St. Louis, MO, USA). After acclimatization, WKY (*n* = 40) rats were randomly divided into five groups (*n* = 8/group) and fed an atherogenic diet (with 0.5% cholesterol and 0.05% cholic acid) and atherogenic diets supplemented with 1% black rice extract (BRF) in the presence or absence of 2% wood sterols (WS) for 11 weeks. The BRF was obtained from Guangdong Province, China. The basic chemical composition of BRF is shown in Table 3 and Figure 2. The WS was obtained from Forbes Medi-Tech Inc. (Vancouver, BC, Canada), previously prepared from tall oil soaps via a proprietary extraction and purification protocol under the GMP food standards. The purity of the final product was over 99% and contained a mixture of sterols and stanols (Table 4 and Figure 1).

The composition of experimental diets is shown in Table 5. Experimental diets were prepared based on an AIN-76A diet [56]. The basal (g/kg) diet contained 250-g/kg casein (ICN Biochemical, Cleveland, OH, USA), 470-g/kg cornstarch (Neptune Food Service, Richmond, BC, Canada), 50-g/kg alphacel nondigestible fiber (ICN Biochemical, Cleveland, OH, USA), 30-g/kg sucrose (Neptune Food Service, Richmond, BC, Canada), 30-g/kg mineral mix (ICN Biochemical, Cleveland, OH, USA), 30-g/kg vitamin mix (ICN Biochemical, Cleveland, OH, USA), 2.0-g/kg choline chloride (ICN Biochemical, Cleveland, OH, USA), 3.0-g/kg d-l methionine (ICN Biochemical, Cleveland, OH, USA), 30-g/kg monofos (Van Waters and Rogers, Abbotsford, BC, Canada), 0.5-g/kg cholic acid (ICN Biochemical Cleveland, OH, USA), 5.0-g/kg cholesterol (ICN Biochemical, Cleveland, OH, USA), 30-g/kg butter (Dairyworld Foods, Burnaby, BC, Canada), and 70-g/kg canola oil (Neptune Food Service, Richmond, BC, Canada) to provide an adequate supply of essential fatty acids.

Animals were individually housed in stainless-steel cages under 25 °C and lighting (14:10 light: dark cycle) conditions and fed diets for 12 h per day for 11 weeks. Daily feed intakes and the weekly body weight gains of animals were routinely recorded throughout the experimental period. Animal care protocols were in accordance with the principles of the Guide to the Care and Use of Experimental Animals, Vol. 1 of the Council of Animal Care [57], as approved by the University of British Columbia Animal Care Committee (H04-0095: 05/02/11).

### 4.2. Experimental Design and Plasma Analysis

At 16 weeks of age, nonfasted rats were killed by exsanguination under halothane anesthesia at 13:00 h, and blood was drawn into heparinized tubes prior to centrifugation for recover plasma (1000× *g* for 5 min, 4 °C). Aliquots of plasma were analyzed for hemoglobin (Cayman Chemical, Ann Arbor, MI, USA), total cholesterol, HDL cholesterol, triacylglycerol, phospholipids glucose, and CRP using commercial kits from Sigma Aldrich (Sigma, St. Louis, MO, USA). HDL was recovered by precipitating non-HDL particles with 0.5-mM sodium phosphotungstic acid and 25-mM magnesium choride. After centrifugation, the supernatant containing the HDL particles was analyzed for cholesterol and triacylglyceride using CHOD-PAP and GPO-PAP, respectively (Boehringer, Mannheim, FRG). The antioxidant capacity of the plasma was measured with the method described by Kitts and Hu [58].

### 4.3. Liver, Heart, and Aorta Tissue Analyses

Liver, heart, and aorta tissues were removed, washed in 4 °C 0.9% NaCl, dried, and weighed. The arotic tissue was removed from the bachicephalic arteries to the bifurcation and the aorta to the iliac branching. Lipids were extracted using the Folch method [59]. Crude lipids content was measured with the Folch method under nitrogen with subsequent drying at 105 °C. The levels of total cholesterol and triacylglycerol were measured using the methods described by Carlson and Goldfarb [60].

### 4.4. Statistics

All data are expressed as mean ± SEM. A paired *t*-test was used to determine if a difference existed between animals fed on the noncholesterol (NC diet) diet and the positive control, atherogenic-formulated (PCCh) diet (*p* < 0.05). One-way analysis of variance (ANOVA; SPSS/Win Inc., IBM Co., Armonk N.Y. USA) was used to test for differences between experimental treatments (PCCh-). Where differences did exist, the source of the differences was identified by the Student–Newman–Keuls multiple range test at a *p* ≤ 0.05 significance level.

## 5. Conclusions

In this study, we presented the effects of known bioactive phytochemical components—namely, an anthocyanin-rich BRF, standardized mixture of WS, and a combination of both—to control hyperlipidemia in rats fed on an atherogenic diet. Feeding WS alone produced lower plasma cholesterol concentrations that were related to lower tissue cholesterol notably in liver and aorta tissues. Similar benefits in controlling plasma and tissue TAGs were observed in rats fed BRF. The combination of adding both WS and BRF to the atherogenic diet also produced an improvement in both cholesterols: HDL cholesterol and non-HDL cholesterol, and TAGs, which were attributed more to the presence of WS or BRF, respectively, than the combination of both. These findings support developing novel diets containing value-added BRF and WS components to enable benefits associated with reduced modifiable atherosclerosis risk factors attributed to hyperlipidemia.

## Figures and Tables

**Table 1:** Lipid profile in the plasma of rats fed experimental diets.

Diet Treatment	TC	HDL	Non-HDL	TAG	PL
NC	1.75 ± 0.10 ^+^	1.12 ± 0.03 ^+^	0.67 ± 0.09 ^+^	0.52 ± 0.04	111.8 ± 9.0
PCCh	4.21 ± 0.11 ^a^	0.74 ± 0.03 ^a^	3.47 ± 0.12 ^a^	0.56 ± 0.03 ^a^	96.97 ± 4.67 ^a^
PCCh + BRF	2.67 ± 0.12 ^b^	0.74 ± 0.06 ^a^	1.74 ± 0.13 ^b^	0.45 ± 0.04 ^b^	89.78 ± 2.1 ^a^
PCCh + WS	2.07 ± 0.06 ^c^	1.08 ± 0.05 ^b^	1.05 ± 0.12 ^c^	0.74 ± 0.03 ^c^	115 ± 4.6 ^b^
PCCh + WS + BRF	2.08 ± 0.07 ^c^	0.73 ± 0.08 ^a^	1.33 ± 0.14 ^c^	0.47 ± 0.02 ^b^	112.5 ± 5.7 ^b^

TC—total cholesterol (mmol/L), HDL—high-density lipoprotein cholesterol (mmol/L), Non-HDL cholesterol (mmol/L), TAG—triacylglycerides (mmol/L), and PL—phospholipids (mg/dL). NC: AIN-76A diet. PCCh: positive control (0.5% cholesterol, 0.05 cholic acid, 3% butter). BRF: black rice fraction. PCCh + BRF: positive control with black rice fraction. PCCh + WS: positive control with wood sterols. PCCh + WS + BRF: positive control with wood sterols and black rice fraction. Data are expressed as means ± SEM (*n* = 8). The + symbol signifies a significant difference (*p* < 0.05) between the negative cholesterol (NC) and positive control group (PCCh). Values in columns with different superscript letters (a, b, and c) are significantly different (*p* ≤ 0.05).

**Table 2:** Lipid concentration in selected tissues in experimental rats (mg/g).

Diet Treatment	Liver CL	Liver TC	Liver TAG	Heart TC	Aorta TC
NC	58 ± 2.83 ^+^	6.19 ± 0.59 ^+^	0.53 ± 0.33 ^+^	0.157 ± 0.01 ^+^	1.14 ± 0.03 ^+^
PCCh	390 ± 20 ^a^	15.92 ± 0.56 ^a^	9.53 ± 0.64 ^a^	0.256 ± 0.03 ^a^	5.86 ± 0.83 ^a^
PCCh + BRF	280 ± 12 ^b^	11.86 ± 1.36 ^b^	6.29 ± 0.95 ^b^	0.186 ± 0.03 ^b^	2.14 ± 0.20 ^b^
PCCh + WS	96 ± 15 ^c^	8.30 ± 0.58 ^c^	10.17 ± 0.50 ^a^	0.245 ± 0.01 ^a^	0.55 ± 0.10 ^d^
PCCh + WS + BRF	120 ± 10 ^c^	8.17 ± 0.71 ^c^	7.73 ± 0.56 ^b^	0.234 ± 0.02 ^a^	1.51 ± 0.15 ^c^

CL = crude lipid (mg/g ww), TC = total cholesterol (mg/g ww), and TAG = triacylglyceride (mg/g ww). NC-AIN-76A diet. PCCh: positive control (0.5% cholesterol, 0.05 cholic acid, 3% butter). BRF:black rice fraction. PCCh + BRF: positive control with black rice fraction. PCCh + WS: positive control with wood sterols. PCCh + WS + BRF: positive control with wood sterols and black rice fraction. Data are expressed as means ± SEM (*n* = 8). The + symbol signifies a significant difference (*p* < 0.05) between the negative cholesterol (NC) and positive control group (PCCh). Values in columns with different superscript letters (a, b, and c) are significantly different (*p* ≤ 0.05).

**Table 3:** Basic chemical composition of the black rice fraction and selected bioactive contents.

Ingredient	g/100 g d.m.
Protein	17
Fat	15
Ash	8.3
Digestible carbohydrates	50.02
Soluble dietary fiber	3.08
Insoluble dietary fiber	6.60
Anthocyanins (%)	
cyanidin-3-*O*-glucoside	97.86
peonidin-3-*O*-glucoside	2.14
Total anthocyanins (mg/g)	31.3
Oryzanol (% of total fat)	3.83

**Table 4:** Wood sterols composition (%).

Total Sterol Content	99.6
Sitostanol	16
Sitosterol	73
Campasterol	6
Stigmasterol	1
Other sterols	2

**Table 5:** Experimental diet compositions (g/100 g).

Ingredient	Negative Control (NC)	PCCh	PCCh + BRF	PCCh + WS	PCCh + WS + BRF
Casein	25.5	25.5	25.5	25·5	25.5
Corn starch	47	45.95	45.5	43.5	43.5
Sucrose	3.0	3.0	3.0	3.0	3.0
Cellulose	5.0	5.0	5.0	5.0	5.0
Monophosphate	3.0	3.0	3.0	3.0	3.0
Vitamin mix	3.0	3.0	3.0	3.0	3.0
Choline chloride	0.20	0.20	0.20	0.20	0.20
D-L Methionine	0.30	0.30	0.30	0.30	0.30
Mineral mix	3.0	3.0	3.0	3.0	3.0
Canola Oil	10	7.0	7.0	7.0	7.0
Butter	-	3.0	3.0	3.0	3.0
Wood sterols	0	-	-	2.0	2.0
Cholesterol	0	0.50	0.50	0.50	0.50
Cholic acid	0	0.050	0.05	0.05	0.05
BRF	-	-	1.0	-	1.0

NC: cholesterol and bile acid-free diet; negative control. PCCh: positive control (0.5% cholesterol, 0.05 cholic acid, 3% butter). BRF: black rice fraction. PCCh + BRF: positive control, atherogenic diet with added black rice fraction. PCCh + WS: positive control, atherogenic diet with added wood sterols. PCCh + WS + BRF: positive control, atherogenic diet with added wood sterols and black rice fraction.

## Supplementary Materials

The following are available online. Table S1: Body gain, heart, liver, weight, hematocrit, and hemoglobin in rats.

## References

[1] Roger V.L., Go A.S., Lloyd-Jones D.M., Adams R.J., Berry J.D., Brown T.M., Carnethon M.R., Dai S., de Simone G., Ford E.S. (2011). Heart Disease and Stroke Statistics—2011 Update. A Report from the American Heart Association. Circulation.

[2] World Health Organization. (WHO) (2018). Noncommunicable Diseases Country Profiles 2018.

[3] Prabhakaran D., Jeemon P., Roy A. (2016). Cardiovascular diseases in India: Current epidemiology and future directions. Circulation.

[4] Li Y., Wang D.D., Ley S.H., Green-Howard A., He Y., Lu Y., Danaei G., Hu F.B. (2016). Potential impact of time trend of life-style factors on cardiovascular disease burden in China. J. Am. Col. Cardiol..

[5] World Health Organization. (WHO) (2017). Global Health Observatory (GHO) Data: Raised Cholesterol. Situation and Trends. https://www.who.int/gho/ncd/risk_factors/cholesterol_text/en/.

[6] Gylling H., Plat J., Turley S., Ginsberg H.N., Ellegård L., Jessup W., Jones P.J., Lütjohann D., Maerz W., Masana L. (2014). Plant sterols and plant stanols in the management of dyslipidaemia and prevention of cardiovascular disease. Atherosclerosis.

[7] Rocha V.Z., Ras R.T., Gagliardi A.C., Mangili L.C., Trautwein E.A., Santos R.D. (2016). Effects of phytosterols on markers of inflammation: A systematic review and meta-analysis. Atherosclerosis.

[8] Jones P.J.H., Shamloo M., MacKay D.S., Rideout T.C., Myrie S.B., Plat J., Roullet J.-B., Baer D.J., Calkins K.L., Davis H.R. (2018). Progress and perspectives in plant sterol and plant stanol research. Nutr. Rev..

[9] Xia M., Ling W.H., Kitts D.D., Zawistowski J. (2003). Supplementation of diets with black rice pigment fraction attenuates atherosclerotic plaque formation in apolipoprotein E deficient mice. J. Nutr..

[10] Hu C., Zawistowski J., Ling W., Kitts D.D. (2003). Black rice (Oryza *sativia* L. *indica*) pigmented fraction suppresses both reactive oxygen species and nitric oxide in chemical and biological model system. J. Agric. Food Chem..

[11] Yousuf B., Gul K., Abas Wani A., Singh P. (2016). Health benefits of anthocyanins and their encapsulation for potential use in food systems: A review. Crit. Rev. Food Sci. Nutr..

[12] Wallace T.C., Slavin M., Frankenfeld C.L. (2016). Systematic review of anthocyanins and markers of cardiovascular disease. Nutrients.

[13] Cabral C.E., Klein M.R.S.T. (2017). Phytosterols in the treatment of hypercholesterolemia and prevention of cardiovascular diseases. Arq. Bras. Cardiol..

[14] Maki K.C., Lawless A.L., Reeves M.S., Kelley K.M., Dicklin M.R., Jenks B.H., Shneyvas E., Brooks J.R. (2013). Lipid effects of a dietary supplement softgel capsule containing plant sterols/stanols in primary hypercholesterolemia. Nutrition.

[15] Jaceldo-Siegel K., Lütjohann D., Sirirat R., Mashchak A., Fraser G.E., Haddad E. (2017). Variations in dietary intake and plasma concentrations of plant sterols across plant-based diets among North American adults. Mol. Nutr. Food Res..

[16] Vanstone C.A., Raeini-Sarjaz M., Parson W.E., Jones P.J.H. (2002). Unesterified plant sterols and stanols lower LDL-cholesterol concentrations equivalently in hypercholesterolemic persons. Am. J. Clin. Nutr..

[17] Rouyanne T., Ras R.T., Geleijnse J.M., Trautwein E.A. (2014). LDL-cholesterol-lowering effect of plant sterols and stanols across different dose ranges: A meta-analysis of randomized controlled studies. Br. J. Nutr..

[18] Kalliny S., Zawistowski J., Aluko R., Birch E.J., Larsen D., Melton L., Rogers M., Shahidi F., Stadler R., Sun-Waterhouse D., Varelis P. (2019). Phytosterols and phytosterols. Encyclopedia of Food Chemistry.

[19] Wilson T.A., Idreis H.M., Taylor C.M., Nicolosi R.J. (2002). Whole fat rice bran reduces the development of aortic atherosclerosis in hypercholesterolemic hamsters compared with wheat bran. Nutr. Res..

[20] Ausman L.M., Rong N., Nicolosi R.J. (2005). Hypocholesterolemic effect of physically refined rice bran oil: Studies of cholesterol metabolism and early atherosclersis in hyprcholesterolemic hamsters. J. Nutr. Biochem..

[21] Zawistowski J., Kopeć A., Kitts D.D. (2009). Effect of a black rice extract (*Oryza sativa* L. *indica*) on cholesterol levels and plasma lipid parameters in Wistar Kyoto rats. J. Funct. Foods.

[22] Ling W.H., Cheng Q.X., Ma J., Wang T. (2001). Red and black rice decrease atherosclerotic plaque formation and increase antioxidant status in rabbits. J. Nutr..

[23] Xia X., Ling W.H., Ma J., Xia M., Hou M., Wang Q., Zhu H. (2006). An anthocyanin-rich extract from black rice enhances atherosclerosis plaque stabilization in apolipoprotein E-deficient mice. J. Nutr..

[24] Novotny J.A., Baer D.J., Khoo K., Gebauer S.K., Charron C.S. (2015). Cranberry juice consumption lowers markers of cardiometabolic risk, including blood pressure and circulating c-reactive protein, triglyceride, and glucose concentrations in adults. J. Nutr..

[25] Hou Z., Qin P., Zhang Y., Cui S., Ren G. (2013). Identification of anthocyanins isolated from black rice (Oryza sativa L.) and their degradation kinetics. Food Res. Int..

[26] Graf D., Seifert S., Jaudszus A., Bub A., Watzl B. (2013). Anthocyanin-rich juice lowers serum cholesterol, leptin and resistin and improves plasma fatty acid composition in Fischer rats. PLoS ONE.

[27] Ling W.H., Wang L.L., Ma J. (2002). Supplementation of the black rice outer layer fraction to rabbits decreases atherosclerotic plaque formation and increases antioxidant status. J. Nutr..

[28] Yang Y., Andrews M.C., Hu Y., Wang D., Qin Y., Zhu Y., Ni H., Ling W. (2011). Anthocyanin extract from black rice significantly ameliorates platelet hyperactivity and hypertriglyceridemia in dyslipidemic rats induced by high fat diets. J. Agric. Food. Chem..

[29] Guo H., Ling W., Wang Q., Liu C., Hu Y., Xia M., Feng X., Xia X. (2007). Effect of anthocyanin-rich extract from black rice (Oryza sativa L. indica) on hyperlipidemia and insulin resistance in fructose-fed rats. Plant Foods Human Nutr..

[30] Jang H.H., Park M.Y., Kim H.W., Leen Y.M., Hwang K.A., Park J.H., Park D.S., Kwon O. (2012). Black rice (Oryza sativa L.) extract attenuates hepatic steatosis in C57BL/6 J mice fed a high-fat diet via fatty acid oxidation. Nutr. Metab..

[31] Pritchard P.H., Li M., Zamfir K., Lukic T., Novak E., Moghadasin M. (2003). Comparison of cholesterol-lowering efficacy and anti-atherogenic properties of hydrogenated versus non-hydrogenated (Phytrol^TM^) tall oil-derived phytrosterols in apo E-deficient mice. Cardiovasc. Drugs Ther..

[32] Fang N., Yu S., Bader T. (2003). Characterization of triterpene alkohol and serol ferulates in rice bran using LC-MS/MS. J. Agric. Food Chem..

[33] He W.S., Zhu H., Chen Z.Y. (2018). Plant Sterols: Chemical and enzymatic structural modifications and effects on their cholesterol-lowering activity. J. Agric. Food Chem..

[34] Redan B.W., Albaugh G.P., Charron C.S., Novotny J.A., Ferruzzi M.G. (2017). Adaptation in Caco-2 human intestinal cell differentiation and phenolic transport with chronic exposure to blackberry (*Rubus sp*) extract. J. Agric. Food Chem..

[35] Wu X., Pittman H.E., Prior R.L. (2006). Fate anhocyanins and antioxidant capacity in contents of the gastrointestinal tract of weanling pigs following black raspberry consumption. J. Agric. Food Chem..

[36] Elisia I., Kitts D.D. (2008). Anthocyanins inhibit peroxyl radical-induced apoptosis in Caco-2 cells. Mol. Cell Biochem..

[37] Vaskonsen T., Mervaala E., Sumuvuori V., Seppän-Laakso T., Karppanen H. (2002). Effect of calcium and plant sterols on serum lipids in obese Zucker rats on a low-fat diet. Br. J. Nutr..

[38] Devaraj R., Autret B.C., Jialal I. (2006). Reduced-calorie orange juice beverage with plant sterols lowers C-reactive protein concentrations and improves the lipid profile in human volunteers. Am. J. Clin. Nutr..

[39] AbuMweis S.S., Vanstone C.A., Ebine N., Kassis A., Ausman L.M., Jones P.J.H., Lichtenstein A.H. (2006). Intake of single morning dose of standard and novel plant sterol preparations for 4 weeks odes not dramatically affected plasma lipid concentratins in humans. J. Nutr..

[40] Fumeron F., Bard J.M., Lecerf J.M. (2017). Interindividual variability in the cholesterol-lowering effect of supplementation with plant sterols or stanols. Nutr. Rev..

[41] Calpe-Berdiel L., Escola-Gil J.C., Blanco-Vaca F. (2006). Phytosterol-mediated inhibition of intestinal cholesterol absorption is independent of ATP-binding cassette transporter A1. Br. J. Nutr..

[42] He J., Giusti M.M. (2010). Anthocyanins: Natural colorants with health-promoting properties. Annu. Rev. Food Sci. Technol..

[43] Reis J.F., Monteiro V.V.S.M., de Souza Gomes R., do Carmo M.M., da Costa G.V., Ribera P.C., Chagas Monteiro M. (2016). Action mechanism and cardiovascular effect of anthocyanins: A systematic review of animal and human studies. J. Transl. Med..

[44] Amiot M.J., Riva C., Vinet A. (2016). Effects of dietary polyphenols on metabolic syndrome features in humans: A systematic review. Obes. Rev..

[45] Pojer E., Mattivi F., Johnson D., Stockley C.S. (2013). The case for anthocyanin consumption to promote human health: A review. Comp. Rev. Food Sci. Food Saf..

[46] Pong C.H., Liu L.K., Chuang C.M., Chyau C.C., Huang C.N., Wang C.J. (2011). Mulberry water extracts possess an anti-obesity effect and ability to inhibit hepatic lipogenesis and promote lipolysis. J. Agric. Food Chem..

[47] Mekki N., Dubois C., Charbonier M., Cara L., Senft M., Pauli A.M., Portugal H., Gassin A.M., Lafon H., Lairon D. (1997). Effects of lowering fat and increasing dietary fiber on fasting and postprandial plasma lipids in hypercholsterolemic subjects consuming a mixed Mediterranean-Western diet. Am. J. Clin. Nutr..

[48] Behall K.M., Scholfield D.J., Hallfrisch J. (2004). Diets containing barley significantly reduce lipids in mildly hypercholesterolemic men and women. Am. J. Clin. Nutr..

[49] Kontush A. (2014). HDL-mediated mechanisms of protection in cardiovascular disease. Cardiovasc. Res..

[50] Morgan A.E., Mooney K.M., Wilkinson S.J., Pickles N.A., Mc Auley M.T. (2016). Cholesterol metabolism: A review of how ageing disrupts the biological mechanisms responsible for its regulation. Aging Res. Rev..

[51] Qin Y., Xia M., Ma J., Hao Y.T., Liu J., Mou H.Y., Cao L., Ling W.H. (2009). Anthocyanin supplementation improves serum LDL- and HDL-cholesterol concentrations associated with the inhibition of cholesteryl ester transfer protein in dyslipidemic subjects. Am. J. Clin. Nutr..

[52] Roohinejad S., Omidizadeh A., Mirhosseini H., Saari N., Mustafa S., Yusof R.M., Hussin A.S.M., Hamid A., Yazid M., Manap A. (2010). Effect of pre-germination time of brown rice on serum cholesterol levels of hypercholesterolaemic rats. J. Sci. Food Agric..

[53] Libby P., Ridker M.P., Hansson G.K. (2011). Progress and challenges in transating the biology of artherosclerosis. Nature.

[54] Ma Y., Griffith J.A., Chasan-Taber L., Olendzki B.C., Jackson E., Stanek E.J., Li W., Pagoto S.L., Hafner A.R., Ockene I.S. (2006). Association between dietary fiber and serum C-reactive protein. Am. J. Clin. Nutr..

[55] Oi L., van Dam R.M., Liu S., Franz M., Mantzoros C., Hu F.B. (2006). Whole-grain, bran, and cereal fiber intakes and markers of systemic inflammation in diabetic women. Diabetes Care.

[56] Reeves P.G., Nielsen F.H., Fahey G.C. (1993). AIN-93 purified diets for laboratory rodents: Final report of the American Institute of Nutrition Ad Hoc Writing Committee on the Reformulation of the AIN-76A Rodent Diet. J. Nutr..

[57] Canadian Council of Animal Care (1983). Guide to the Care and Use of Experimental Animals.

[58] Kitts D.D., Hu C. (2005). Biological and chemical assessment of antioxidant activity of sugar-lysine model Maillard Reaction Products. Ann. N. Y. Acad. Sci..

[59] Folch J., Lees M., Sloane-Stanley G.H. (1957). A simple method for the isolation and purification of total lipids from animal tissues. J. Biol. Chem..

[60] Carlson S.E., Goldfarb S. (1979). A sensitive enzymatic method for the determination of free and esterified tissue cholesterol. Clin. Chim. Acta.

