# Supplementary material for: Benefits of Anthocyanin-Rich Black Rice Fraction and Wood Sterols to Control Plasma and Tissue Lipid Concentrations in Wistar Kyoto Rats Fed an Atherogenic Diet
Source: Molecules. 2020 Nov 17;25(22):5363. doi: 10.3390/molecules25225363 (PMC7698440; doi:10.3390/molecules25225363)
Supplement: Supplementary file 1 [file molecules-25-05363-s001.pdf]

Table S1. Food intake during experiment, heart and liver, weight, hematocrit and hemoglobin in rats.

Treatment	Food intake [g]	Heart weight [g]	Liver weight [g]	Hematocrit [%]	Hemoglobin mg/mL
NC	615±20	1.07±0.04	12.62±0.33	0.87±0.02[+]	11.5±0.25
PCCh	416±25[a]	1.13±0.05[a]	19.99±0.85[a]	0.41±0.02[a]	11.7±0.270[a]
PCCh+BRF	472±15[b]	1.10±0.04[a]	17.96±0.99[b]	0.38±0.01[a]	11.0±0.32[a]
PCCh+WS	445±21[a]	1.06±0.03[a]	13.55±0.45[c]	0.40±0.08[a]	11.1±0.33[a]
PCCh+WS+BRF	414±17[b]	1.08±0.04[a]	13.72±0.55[c]	0.42±0.01[a]	11.2±0.25[a]

NC- AIN-76A diet

PCCh positive control (0.5% cholesterol, 0.05 cholic acid, 3% butter)

BRF-black rice fraction

PCCh/PC+BRF positive control with black rice fraction

PCCh/PC+WS positive control with wood sterols,

PCCh/PC+WS+BRF positive control with wood sterols and black rice fraction

Data are expressed as means ± SEM (n=8). The symbol + signifies a significant difference (P<0.05) between the negative cholesterol (NC) and positive control group (PCCh); values in column with different letter (a, b, c) are significantly different at P≤0.05.
